# Application of Endoscopic Ultrasound Combined with Multislice Spiral CT in Diagnosis and Treatment of Patients with Gastrointestinal Eminence Lesions

**DOI:** 10.1155/2022/1417104

**Published:** 2022-06-29

**Authors:** Jie Xiong, Jie Jiang, Ying Chen, Ye Chen, Chenyi Xie, Shuchang Xu

**Affiliations:** ^1^Department of Gastroenterology and Hepatology, Tongji Hospital, Tongji University School of Medicine, Shanghai 200065, China; ^2^National Center for Liver Cancer, Second Military Medical University, Shanghai 200438, China; ^3^Center of Minimally Invasive Treatment for Tumor, Department of Medical Ultrasound, Shanghai Tenth People's Hospital, Tongji University School of Medicine, Shanghai 200072, China

## Abstract

**Objective:**

To evaluate the application of endoscopic ultrasound (EUS) combined with multislice spiral CT (MSCT) in the diagnosis and treatment of patients with gastric eminence lesions.

**Methods:**

A total of 160 patients with gastric eminence lesions enrolled in our hospital from June 2018 to June 2021 were included and received EUS and MSCT. The results of the two examinations and the postoperative pathological results were compared.

**Results:**

The common pathological types of gastric eminence lesions include polyps and stromal tumors, with the most common sites of lesions in the gastric antrum, followed by the fundus of the stomach and the gastric body. Gastric eminence lesions mostly originate from the mucosal layer and muscularis mucosa, accounting for 83.13% of the total. With pathological results as the gold standard, the detection rate of MSCT was 90.63%, and that of EUS was 78.13%. With the joint diagnosis as a reference, the receiver operating curve (ROC) revealed a higher diagnostic efficiency of MSCT and EUS.

**Conclusion:**

The accuracy of MSCT in the diagnosis of gastric eminence lesions is significantly higher than that of EUS, both of which can offer useful guidance for the choice of endoscopic treatment methods. The combination of MSCT and EUS examination before endoscopic gastroscopy may provide a better treatment efficacy on gastric protruding lesions with high safety.

## 1. Introduction

Gastric eminence lesions are mostly caused by the compression of extramural organs or lesions and the large folds of the gastric mucosa [1–3]. Gastric eminence lesions are caused by the degeneration and necrosis of the vagus nerve and sympathetic nerve, resulting in abnormal secretion of various intestinal hormones and changes in the intestinal environment [4]. Currently, the diagnostic efficiency of the property and origin of the lesion by electronic gastroscopy is poor, so endoscopy and multislice spiral CT (MSCT) are frequently used in clinical practice for preoperative diagnosis to provide a guidance for subsequent treatment [5–8]. Endoscopic ultrasound (EUS), with ultrasound and endoscopy, can clearly show the digestive tract wall, adjacent organs, and tissues, which helps clarify the cause of the lesion by preliminarily determining the property of the lesion according to its echo characteristics [9–12]. MSCT can clearly show the depth, location, and size of tumor infiltration and can effectively determine the presence of lymph node metastasis in the lung, mediastinum, and distal organ tissues. EUS allows direct visualization of mucosal lesions in the gastrointestinal tract, while real-time scanning can be performed using endoscopic ultrasound, thus further improving diagnostic accuracy. To further enhance the treatment efficacy of gastric eminence lesions this study explored the application value of EUS combined with MSCT in the diagnosis of patients with gastric eminence lesions to provide a clinical reference of endoscopic treatment.

## 2. Research Design

### 2.1. Patient Screening and Grouping

A total of 160 patients with gastric eminence lesions enrolled in Tongji Hospital, Tongji University School of Medicine, from June 2018 to June 2021 were included for retrospective analysis. This study has been approved by the ethics committee of Tongji Hospital, Tongji University School of Medicine, No. 197TJ29-1.

### 2.2. Inclusion Criteria

Patients who were diagnosed with gastric eminence lesions after receiving electronic gastroscopy in our hospital and received endoscopic treatment after further determination of the range and property of the lesions by EUS and MSCT, with complete clinical data, and who were fully informed of the purpose and process of the study and provided written informed consent were included.

### 2.3. Exclusion Criteria

Patients with progressive gastric cancer; with external pressure lesions; with other serious organic diseases, coagulation dysfunction, or malignant tumors; and with cognitive impairment, communication impairment, or physical impairment were excluded.

### 2.4. Methods

*EUS examination*: the examination was carried out using an ultrasound host (model: Fuji SU-8000) + EUS (model: Fuji EG-530UT), equipped with 12, 15, and 20 MHz ultrasound probes. Small superficial lesions were inspected with the 12 MHz ultrasound microprobe. The water filling method and the water bag technique were used, and the frequency and scanning method of the ultrasound probe was switched according to the size and location of the lesion [13–16].

*MSCT examination*: the patients fasted for 8 hours, drank 600-1000 ml of water 20 minutes before the examination, and received 20 mg of amidoamine (Fujian Sanai Pharmaceutical Co., Ltd., Approval No. H35020158) through intramuscular injection. With the patients in a supine position according to the conditions of the gastric lesions, a plain CT scan was performed, ranging from the right side of the diaphragm to the duodenum, with a voltage of 120 KV, a current of 250-300 mA, a thickness of 5 mm, a pitch of 1.25, and reconstruction thickness of 0.625 mm [17–20]. Then, contrast CT was performed after the injection of the iopromide (Schering Pharmaceutical Co., Ltd., Approval No. H10970166) using a high-pressure syringe, with an injection speed of 3.5 ml/s. Arterial phase was scanned 30 s after injection covering the esophagus, abdomen, and whole stomach, and venous phase was scanned 60 s after the injection to observe the tissue damage adjacent to the lesion and the liver and distant metastasis.

The imaging examination results of all patients were diagnosed by two radiologists and sonographers.

Endoscopic treatment was performed for some eminence lesions, including high-frequency electrocoagulation resection, mucosal resection, mucosal dissection-tumorectomy, mucosal dissection-tumor excision plus titanium clip closure, and puncture sclerotherapy; some submucosal tumors were surgically removed, and pathological examination was performed. Mucosal dissection-tumor tumorectomy uses a double-port therapeutic endoscope. A snare is placed through one port to cover the mucosal tissue on the tumor surface, and high-frequency electrical resection of the surface mucosa is performed, or a needle knife is used to cut the mucosal tissue on the surface of the tumor to expose the submucosal tumor; then, a biopsy forceps is used through another hole to clamp the tumor and lift it up, and a snare is used to cover the tumor at the bottom of the tumor, to perform high-frequency electrical resection.

### 2.5. Postoperative Follow-Up

The patients received reexamination by gastroscopy at 3, 6, and 12 months after surgery to obtain the patient recovery data. Biopsy was performed again to determine the occurrence of local recurrence if necessary.

### 2.6. Statistical Analyses

The data obtained in this study were analyzed using SPSS20.0 software, and GraphPad Prism 7 (GraphPad Software, San Diego, USA) was used to plot the graphics. The counting data are expressed as *n*(%) and analyzed using the chi-square test. The measurement data are expressed as mean ± SD and analyzed using the *t*-test. The specificity and sensitivity of receiver operating curve (ROC) and the area under the curve (AUC) were calculated. Differences were considered statistically significant at *P* < 0.05. Sensitivity = number of true positives/(number of true positives + number of false negatives)∗100%; specificity = number of true negatives/(number of true negatives + number of false positives)∗100%. The sensitivity and specificity of this study are for the detection of gastric eminence lesions, with no subdivision of the disease.

## 3. Results

### 3.1. The Distribution of Gastric Eminence Lesions

The distribution of the lesions of 160 patients was analyzed. The diameter of gastric eminence lesions was about 1.47 ± 0.95 cm. The common pathological types of gastric eminence lesions include polyps and stromal tumors, with the most common sites of lesions in the gastric antrum, followed by the fundus of the stomach and the gastric body. The pathological types of the gastric antrum were mostly polyps, of the fundus lesions were mostly stromal tumors, of the gastric body lesions were mainly polyps on the greater curvature side, and of the gastric angle were chiefly malignant (Table 1).

### 3.2. Histological Characteristics of Gastric Eminence Lesions

Gastric eminence lesions mostly originate from the mucosal layer and muscularis mucosa, accounting for 83.13% of the total (Table 2).

### 3.3. Diagnosis Results of Pathology, EUS, and MSCT

With pathological results as the gold standard, the detection rate of MSCT was 90.63%, and that of EUS was 78.13% (*X*^2^ = 9.4815, *P* = 0.005). The preoperative MSCT examination did not provide a clear diagnosis result for 15 patients, with gastric antrum being the most common lesion (40%) and muscularis mucosa being the most common level of lesion causes (46.67%). The preoperative EUS examination did not provide a clear diagnosis result for 35 patients, with gastric antrum being the most common lesion (40%), muscularis mucosa being the most common level of lesion causes (45.71%), and stromal tumor (25.71%) being the most frequent type of lesions (Tables 3–5).

### 3.4. ROC Curve Evaluates the Diagnostic Efficacy of EUS and MSCT

**Figure 1:**
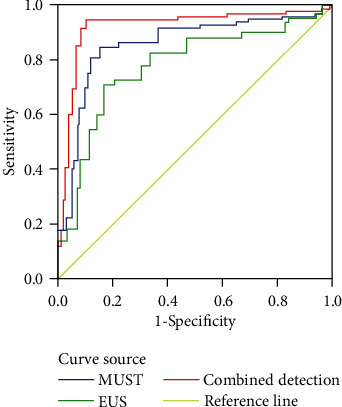
ROC curve.

With the joint diagnosis as a reference, the ROC curve was formulated to compare the diagnostic efficiency: combined detection > MSCT > EUS (*P* < 0.05). The AUC of MSCT was 0.855 (0.850, 0.900), with a sensitivity of 0.806 and specificity of 0.807; the AUC of EUS was 0.871 (0.860, 0.963), with a sensitivity of 0.845 and specificity of 0.811; and the AUC of the combined assay was 0.900 (0.955, 0.999), with a sensitivity of 0.916 and specificity 0.914 (Figure 1).

### 3.5. Treatment Results

Among the 160 patients, 83 patients (77 cases with polyps, 4 cases with stromal tumors, 1 case with malignant lesions, and 1 case with papilloma) underwent high-frequency electrocoagulation resection, and 6 patients (2 cases with polyps, 1 case with stromal tumors, 1 case with adenoma, 1 case with papilloma, and 1 case with cysts) underwent high-frequency electrocoagulation resection, with good healing of the wound 6 months after treatment and no local recurrence after 6-24 months of follow-up. Of 69 patients (2 cases with polyps, 42 cases with stromal tumors, 15 cases with malignant lesions, 4 cases with lipoma, 3 cases with adenoma, 1 case with hemangioma, 1 case with schwannoma, and 1 case with lymphangioma) who received mucosal dissection-tumorectomy, 3 cases experienced intraoperative bleeding, and 2 cases had delayed postoperative bleeding, for which hemostasis was performed successfully. Due to the large tumor body and the risk of perforation, 2 patients (1 case with stromal tumors and 1 case with malignant lesions) were transferred to surgical treatment, in which the tumors were further determined as gastric fundus stromal tumors, with a propensity for malignancy. The 2 patients were lost at postoperative follow-up (see Table 6 for details).

## 4. Discussion

In the diagnosis of gastric eminence lesions, EUS presents the shape and size of the lesion through endoscopy and clearly shows the structure of the stomach wall, from the mucosal layer to the muscularis mucosa, the submucosal layer, the muscularis propria, and the serosal layer, with hyperecho, hypoecho, hyperecho, hypoecho, and hyperecho signals, respectively. It yields high accuracy in determining the size of the lesion and the origin and range of the lesion tissue [21–24].

With pathological results as the gold standard, the detection rate of EUS was 78.13%. 35 patients had unclear EUS results, with gastric antrum being the most common lesion (40%), muscularis mucosa being the most common level of lesion causes (45.71%), and stromal tumor (25.71%) being the most frequent type of lesion, indicating a high rate of missed diagnosis of the stromal tumors, which is consistent with the research results by Ikoma et al. [25]. MSCT can realize multidirectional and multiangle observation of the lesion by using technologies such as MPR, which clearly presents the tumor size, location, shape, growth pattern, boundary, enhancement characteristics, ulcer, and calcification degree and shows the relationship between the disease and surrounding organs and lymph node metastasis, especially for stromal tumors. In the present study, the detection rate of MSCT was 90.63%, which was significantly higher than that of 78.13% for EUS.

The common pathological types of gastric eminence lesions tumor include polyps and stromal tumors, with the most common sites of lesions in the gastric antrum, followed by the fundus of the stomach and the gastric body. Gastric eminence lesions mostly originate from the mucosal layer and muscularis mucosa, accounting for 83.13% of the total. With the joint diagnosis as a reference, the ROC curve revealed a higher diagnostic efficiency of MSCT versus EUS. Clinically, misdiagnosis is frequently seen in stromal tumors as hypoechoic and well-defined masses in EUS. Stromal tumors mostly occur in the fundus of the stomach, which originate in the muscle layer. The signs of MSCT for mesenchymal tumors are intracavernous growth with clear borders and uniform enhancement on enhancement scans when the tumor is small in size and irregular in shape with blurred borders and inhomogeneous enhancement on enhancement scans when the tumor is large in size. In terms of misdiagnosis in the present study, EUS mostly involved stromal tumors, while MSCT had a high diagnostic yield for stromal tumors, suggesting that MSCT plays a certain auxiliary role in distinguishing stromal tumors. The ROC revealed that in the diagnosis of gastric eminence lesions, the performance of a single ultrasound image is undesirable, while the combination of EUS and MSCT is more effective. Furthermore, the combination of clinical manifestations and pathological characteristics of the patients can prominently enhance the accuracy of the diagnosis of lesions.

Among the 160 patients, 83 underwent high-frequency electrosurgery, and 6 underwent nylon thread ligation, with good healing of the wound 6 months after treatment and no local recurrence after 6-24 months of follow-up. Of 69 patients who received mucosal dissection-tumorectomy, 3 cases experienced intraoperative bleeding and 2 cases had delayed postoperative bleeding, for which hemostasis was performed successfully; 2 cases were transferred to surgical treatment. In this study, no cases of perforation were found, which may be attributed to the operation skills or the small sample size of this study, suggesting that mucosal dissection-tumorectomy is safe in the treatment of gastric eminence lesions and can enhance the treatment effect. The microultrasound of ultrasound endoscopy cannot penetrate the intrinsic muscle layer of the tumor and identify the level of lesion, and some patients have peritoneal fibrosis and inflammatory reaction, which may further thicken the esophageal wall and adversely affect the accuracy of staging diagnosis. MSCT is a cross-sectional scan, which cannot effectively distinguish esophageal cancer from the normal esophageal wall and is less effective in identifying soft tissues.

Chinese medicine has accumulated a wealth of experience in the treatment of gastric eminence lesions, which can effectively relieve symptoms and enhance clinical efficacy. Gastric eminence lesions can be treated with catgut embedding acupuncture. The catgut used is an allogeneic protein, and when the allogeneic protein is buried in the acupuncture point, the body's rejection reaction to the allogeneic protein continues to stimulate the acupuncture point, thereby achieving the purpose of treating diseases, with the advantages of economic applicability, simple operation, short duration, and high efficiency.

## 5. Conclusion

The accuracy of MSCT in the diagnosis of gastric eminence lesions is significantly higher than that of EUS, both of which can offer useful guidance for the choice of endoscopic treatment methods. The combination of MSCT and EUS examination before endoscopic gastroscopy may provide a better treatment efficacy on gastric protruding lesions with high safety. The limitation of this study is that less follow-up study was performed in postoperative patients; therefore, this study only serves as a guide for diagnosis, and further research in the treatment is required in future studies.

## Figures and Tables

**Table 1:** The distribution of different pathological types of gastric eminence lesions.

	Polyp	Stromal tumor	Malignant lesions	Lipoma	Adenoma	Papilloma	Hemangioma	Cyst	Schwannoma	Lymphangiomyoma	Total
Cardia	11	0	0	0	0	2	0	0	0	0	15
Fundus of stomach	4	35	0	0	0	0	0	1	0	0	40
Greater curvature of the stomach	5	3	3	0	0	0	0	0	1	0	12
Lesser curvature of the stomach	2	3	0	0	0	0	1	0	0	0	6
Gastric anterior wall	3	2	0	0	1	0	0	0	0	1	7
Gastric posterior wall	3	2	0	1	0	0	0	0	0	0	6
Gastric angle	0	0	3	0	0	0	0	0	0	0	3
Greater curvature of gastric antrum	14	0	6	1	1	0	0	0	0	0	22
Lesser curvature of gastric antrum	13	0	0	0	0	0	0	0	0	0	13
Anterior wall of gastric antrum	13	1	1	2	0	0	0	0	0	0	17
Posterior wall of gastric antrum	11	0	4	0	2	0	0	0	0	0	17
Pylorus	2	0	0	0	0	0	0	0	0	0	2
Total	81	48	17	4	4	2	1	1	1	1	160

**Table 2:** Origin level of different pathological types of gastric eminence lesions.

Type of lesion	Mucosal layer	Muscularis mucosa	Submucosa	Muscularis propria	Unclear origin	Total
Polyp	77	1	1	1	2	81
Stromal tumor	0	42	4	2	0	48
Malignant lesions	0	9	7	1	0	17
Lipoma	0	0	4	0	0	4
Adenoma	0	3	1	0	0	4
Papilloma	0	1	1	0	0	2
Hemangioma	0	0	0	0	0	1
Cyst	0	0	1	0	0	1
Schwannoma	0	0	1	0	0	1
Lymphangiomyoma	0	0	0	1	0	1
Total	77	56	20	5	2	160

**Table 3:** Diagnosis results of pathology, EUS, and MSCT.

Type of lesion	Pathological results	MSCT	EUS	MSCT and EUS
Polyp	81	72	70	81
Stromal tumor	48	44	36	48
Malignant lesions	17	14	10	17
Lipoma	4	5	4	4
Adenoma	4	5	2	4
Papilloma	2	2	1	2
Hemangioma	1	1	1	1
Cyst	1	2	1	1
Schwannoma	1	0	0	0
Lymphangiomyoma	1	0	0	0
Total	160	145	125	158

**Table 4:** The pathological condition of undiagnosed patients by MSCT.

Origin level	n	Lesion site	Type of lesion
Muscularis mucosa	7	Antrum (3 cases)	3 cases of polyps
		Fundus of stomach (3 cases)	3 cases of stromal tumor
		Stomach body (1 case)	1 case of stromal tumor

Submucosa	4	Antrum (3 cases)	3 cases of polyps
		Stomach body (1 case)	1 case of gastric mucosa-associated lymphoma

Muscularis propria	3	Stomach body (2 cases)	2 cases of lymphangiomas
		Antrum (1 case)	1 case of polyps

Unclear origin	1	Antrum (1 case)	1 case of polyps

**Table 5:** The pathological condition of undiagnosed patients by EUS.

Origin level	n	Lesion site	Type of lesion
Muscularis mucosa	16	Fundus of stomach (8 cases)	8 cases of stromal tumors
		Antrum (8cases)	8 cases of malignant lesions

Submucosa	12	Antrum (7 cases)	7 cases of lipoma
		Fundus of stomach (4 cases)	4 cases of stromal tumors
		Stomach body (1 case)	1 case of schwannoma

Muscularis propria	5	Stomach body (4 cases)	2 cases of polyp, 1 case of stromal tumors, 2 cases of lymphangioma

Unclear origin	2	Cardia	2 cases of polyps

**Table 6:** Treatment methods for different pathological types of lesions.

Lesion	n	Endoscopic minimally invasive treatment	
		High-frequency electrocoagulation resection	Mucosal dissection-tumorectomy
Polyp	81	79	2
Stromal tumor	48	5	43
Malignant lesions	17	1	16
Lipoma	4	0	4
Adenoma	4	1	3
Papilloma	2	2	0
Hemangioma	1	0	1
Cyst	1	1	0
Schwannoma	1	0	1
Lymphangioma	1	0	1
Total	160	89	71

## Data Availability

The datasets used and/or analyzed during the current study are available from the corresponding author on reasonable request.
